# [μ-2,2,4,4,6,6-Hexakis(3,5-dimethyl­pyrazol-1-yl)-2λ^5^,4λ^5^,6λ^5^-1,3,5,2,4,6-triaza­triphosphinine]bis­[bis­(nitrato- κ^2^ *O*,*O*′)cadmium(II)]

**DOI:** 10.1107/S160053680803585X

**Published:** 2008-11-08

**Authors:** Sung Yol Yun, Soon W. Lee

**Affiliations:** a Department of Chemistry (BK21), Sungkyunkwan University, Natural Science Campus, Suwon 440-746, South Korea

## Abstract

The complete title complex, [Cd_2_(NO_3_)_4_(C_30_H_42_N_15_P_3_)], is generated by crystallographic twofold symmetry, with one P and one N atom of the cyclo­triphosphazene ligand located on the rotation axis. The non-planar cyclo­triphosphazene ring accommodates two Cd ions, and only four out of six exocylcic pyrazolyl ligands are bound to the Cd metal atoms. Each of these two symmetry-related Cd atoms is coordinated by two bidentate nitrato ligands, two exocylic pyrazolyl N atoms, and one cyclo­triphosphazene N atom.

## Related literature

For background, see: Allen (1991 ▶); Byun *et al.* (1996 ▶); Chandrasekhar & Nagendran (2001 ▶); Mark *et al.* (2005 ▶); Thomas *et al.* (1997 ▶ and references therein). For the synthesis of the ligand, see: Thomas *et al.* (1993 ▶). For related structures, see: Yun & Lee (2008 ▶).
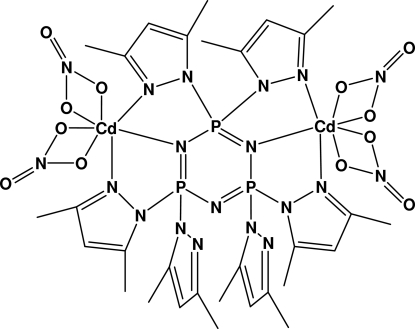


## Experimental

### 

#### Crystal data

[Cd_2_(NO_3_)_4_(C_30_H_42_N_15_P_3_)]
*M* *_r_* = 1178.54
Orthorhombic, 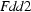

*a* = 28.2418 (5) Å
*b* = 36.2033 (6) Å
*c* = 10.3673 (2) Å
*V* = 10600.0 (3) Å^3^
*Z* = 8
Mo *K*α radiation
μ = 0.96 mm^−1^
*T* = 296 (2) K
0.30 × 0.14 × 0.10 mm

#### Data collection

Bruker SMART CCD diffractometer
Absorption correction: multi-scan (*SADABS*; Bruker, 1997 ▶) *T*_min_ = 0.851, *T*_max_ = 0.908
32426 measured reflections
6260 independent reflections
5122 reflections with *I* > 2σ(*I*)
*R*_int_ = 0.033

#### Refinement

*R*[*F* ^2^ > 2σ(*F* ^2^)] = 0.044
*wR*(*F* ^2^) = 0.112
*S* = 1.04
6260 reflections
299 parameters
1 restraint
H-atom parameters constrained
Δρ_max_ = 0.85 e Å^−3^
Δρ_min_ = −0.48 e Å^−3^
Absolute structure: Flack (1983 ▶), 2758 Friedel pairs
Flack parameter: −0.02 (2)

### 

Data collection: *SMART* (Bruker, 1997 ▶); cell refinement: *SAINT* (Bruker, 1997 ▶); data reduction: *SAINT*; program(s) used to solve structure: *SHELXTL* (Sheldrick, 2008 ▶); program(s) used to refine structure: *SHELXTL*; molecular graphics: *SHELXTL*; software used to prepare material for publication: *SHELXTL*.

## Supplementary Material

Crystal structure: contains datablocks global, I. DOI: 10.1107/S160053680803585X/hb2796sup1.cif

Structure factors: contains datablocks I. DOI: 10.1107/S160053680803585X/hb2796Isup2.hkl

Additional supplementary materials:  crystallographic information; 3D view; checkCIF report

## Figures and Tables

**Table 1:** Selected bond lengths (Å)

Cd1—N1	2.546 (3)
Cd1—N4	2.265 (3)
Cd1—N8	2.311 (4)
Cd1—O1	2.509 (5)
Cd1—O2	2.365 (6)
Cd1—O5	2.367 (8)
Cd1—O4	2.373 (9)

**Fig. 1.**
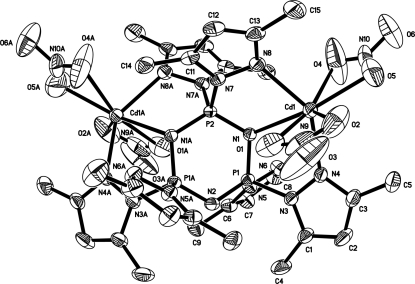
The molecular structure of (I) showing 50% probability displacement ellipsoids. H atoms are omitted for clarity. Atoms with the suffix A are generated by the symmetry operation (–x, –y, z).

## supplementary crystallographic information

### Comment

Polyphosphazenes, linear or cyclic, are an important class of inorganic macromolecules (Mark *et al.*, 2005), and various cyclotriphosphazene derivatives are frequently used as ligands for the preparation of their intriguing coordination and organometallic complexes (Allen, 1991; Chandrasekhar & Nagendran, 2001). Particular attention has been paid to the six-membered cyclotriphosphazene N_3_P_3_(3,5-Me_2_pz)_6_ (3,5-Me_2_pz = 3,5-dimethylpyrazolyl), due to its several potential donor sites such as the exocyxlic pyrazolyl nitrogen atoms and the central cyclotriphosphazene ring nitrogen and phosphorus atoms. This ligand binds to transition metals *via* (1) two non-geminal pyrazolyl N atoms (non-geminal N_2_ coordination), (2) two non-geminal pyrazolyl N atoms and one cyclotriphosphazene ring nitrogen (non-geminal N_3_ coordination), (3) two geminal pyrazolyl N atoms (geminal N_2_ coordination), or (4) two geminal pyrazolyl N atoms and one ring nitrogen (geminal N_3_ coordination) (Thomas *et al.*, 1997). We recently reported the structure of a *C*_3_-symmetric tripalladium–cyclotriphosphazene complex, in which the cyclotriphosphazene exhibits the geminal N_2_ coordination mode (Yun & Lee, 2008). In this paper, we describe the preparation and structure of the title compound, (I), a dicadmium–cyclotriphosphazene complex [Cd_2_(NO_3_)_4_(N_3_P_3_(3,5-Me_2_pz)_6_)].

The molecular structure of (I) is given in Fig. 1, which demonstrates the non-geminal N_3_ coordination mode of the cyclotriphosphazene ligand. This molecule possesses a crystallographic 2-fold axis passing through the P2 and N2 atoms, which explains the *Z* value of 8 instead of 16. The cyclotriphosphazene ring is severely distorted from planarity with an average atomic displacement of 0.146 Å. Each Cd(II) metal is seven-coordinate and bonded to four O atoms from two nitrates, two N atoms from two imidazole rings, and one nitrogen atom from the cyclotriphosphazene ring (Table 1). Four imidazole N atoms coordinate to the two Cd metals to form four 5-membered (PdPN_3_) chelate rings. The fact that the cyclotriphosphazene ring accommodates only two rather than three Cd(NO_3_)_2_ units may be attributed to the steric bulk of the 7-coordinate Cd metals.

The Cd—N_pyz_ bond lengths [2.265 (3)–2.311 (4) Å] are significantly shorter than the Cd—N_ring_ bond length [2.546 (3) Å], indicating that the Cd ions interact more strongly with the imidazole N atoms than with the cyclotriphosphazene ring N atoms. Consistent with our expectation, the average P—N_ring_ bond length [1.583 (3) Å] is considerably shorter than the average P—N_pyz_ bond length [1.677 (3) Å]. The Cd···Cd separation is 7.0177 (6) Å, which is shorter than the corresponding separation (7.195 Å) observed in the chloro analogue [Cd_2_Cl_4_(N_3_P_3_(3,5-Me_2_pz)_6_)] (Byun *et al.*, 1996).

### Experimental

The ligand was prepared by the literature method (Thomas *et al.*, 1993). An acetone (30 ml) solution containing Cd(NO_3_)_2_.4H_2_O (0.144 g, 0.75 mmol) and ligand N_3_P_3_(3,5-Me_2_pz)_6_ (0.176 g, 0.25 mmol) was stirred for 24 h at room temperature. The resulting white solution was filtered off, washed with diethyl ether (6 ml *x* 2) and then hexane (5 ml *x* 2) to give a white solid, which was crystallized from acetone/hexane (1:1 v/v) to yield colorless blocks of (I). IR (KBr, cm^-1^): 2964 (*s*), 2362 (*m*), 1576 (*m*), 1383 (*s*), 1260 (*s*), 1095 (*s*), 1028 (*s*), 805 (*s*). mp: 411–413 K.

### Refinement

The hydrogen atoms were generated in ideal positions (C—H = 0.93–0.96Å) and refined in a riding model.  The nitrato ligands are slightly disordered, but the disorder was not resolved and anisotropic refinement applying several possible site occupation factors was unstable. Site occupancy refinements of the nitrato atoms all yielded values close to unity.

### Crystal data

**Table:**

[Cd_2_(NO_3_)_4_(C_30_H_42_N_15_P_3_)]	*F*_000_ = 4736
*M**_r_* = 1178.54	*D*_x_ = 1.477 Mg m^−^^3^
Orthorhombic, *F**d**d*2	Mo *K*α radiation λ = 0.71073 Å
Hall symbol: F 2 -2d	Cell parameters from 9939 reflections
*a* = 28.2418 (5) Å	θ = 2.2–25.5º
*b* = 36.2033 (6) Å	µ = 0.96 mm^−^^1^
*c* = 10.3673 (2) Å	*T* = 296 (2) K
*V* = 10600.0 (3) Å^3^	BLOCK, colourless
*Z* = 8	0.30 × 0.14 × 0.10 mm

### Data collection

**Table:**

Bruker SMART CCD diffractometer	6260 independent reflections
Radiation source: sealed tube	5122 reflections with *I* > 2σ(*I*)
Monochromator: graphite	*R*_int_ = 0.033
*T* = 296(2) K	θ_max_ = 28.4º
ω scans	θ_min_ = 2.2º
Absorption correction: multi-scan(SADABS; Bruker, 1997)	*h* = −36→28
*T*_min_ = 0.851, *T*_max_ = 0.908	*k* = −48→40
32426 measured reflections	*l* = −13→13

### Refinement

**Table:**

Refinement on *F*^2^	Hydrogen site location: inferred from neighbouring sites
Least-squares matrix: full	H-atom parameters constrained
*R*[*F*^2^ > 2σ(*F*^2^)] = 0.044	*w* = 1/[σ^2^(*F*_o_^2^) + (0.0676*P*)^2^ + 6.1853*P*] where *P* = (*F*_o_^2^ + 2*F*_c_^2^)/3
*wR*(*F*^2^) = 0.112	(Δ/σ)_max_ = 0.001
*S* = 1.04	Δρ_max_ = 0.85 e Å^−^^3^
6260 reflections	Δρ_min_ = −0.48 e Å^−^^3^
299 parameters	Extinction correction: none
1 restraint	Absolute structure: Flack (1983), 2758 Friedel pairs
Primary atom site location: structure-invariant direct methods	Flack parameter: −0.02 (2)
Secondary atom site location: difference Fourier map	

### Special details

**Table:**

Geometry. All e.s.d.'s (except the e.s.d. in the dihedral angle between two l.s. planes) are estimated using the full covariance matrix. The cell e.s.d.'s are taken into account individually in the estimation of e.s.d.'s in distances, angles and torsion angles; correlations between e.s.d.'s in cell parameters are only used when they are defined by crystal symmetry. An approximate (isotropic) treatment of cell e.s.d.'s is used for estimating e.s.d.'s involving l.s. planes.
Refinement. Refinement of *F*^2^ against ALL reflections. The weighted *R*-factor *wR* and goodness of fit *S* are based on *F*^2^, conventional *R*-factors *R* are based on *F*, with *F* set to zero for negative *F*^2^. The threshold expression of *F*^2^ > σ(*F*^2^) is used only for calculating *R*-factors(gt) *etc*. and is not relevant to the choice of reflections for refinement. *R*-factors based on *F*^2^ are statistically about twice as large as those based on *F*, and *R*- factors based on ALL data will be even larger.

### Fractional atomic coordinates and isotropic or equivalent isotropic displacement parameters (Å^2^)

**Table:**

	*x*	*y*	*z*	*U*_iso_*/*U*_eq_	
Cd1	−0.083094 (12)	−0.072056 (9)	0.52427 (3)	0.05498 (12)	
P1	−0.04843 (3)	−0.00189 (2)	0.74616 (9)	0.0339 (2)	
P2	0.0000	0.0000	0.51827 (12)	0.0311 (2)	
O1	−0.0099 (2)	−0.0965 (2)	0.6318 (6)	0.1100 (19)	
O2	−0.0638 (3)	−0.13446 (15)	0.5667 (8)	0.144 (3)	
O3	−0.0064 (4)	−0.1554 (3)	0.6716 (11)	0.251 (7)	
O4	−0.1372 (4)	−0.0530 (3)	0.3622 (15)	0.258 (8)	
O5	−0.1455 (4)	−0.1014 (3)	0.4113 (10)	0.195 (5)	
O6	−0.1902 (3)	−0.0824 (2)	0.2697 (10)	0.170 (4)	
N1	−0.04555 (11)	−0.01150 (8)	0.5958 (3)	0.0357 (7)	
N2	0.0000	0.0000	0.8214 (4)	0.0408 (10)	
N3	−0.08391 (12)	−0.03495 (9)	0.8082 (3)	0.0429 (8)	
N4	−0.10447 (12)	−0.06131 (10)	0.7314 (3)	0.0450 (8)	
N5	−0.07821 (10)	0.03652 (9)	0.7738 (4)	0.0431 (7)	
N6	−0.12225 (13)	0.03751 (11)	0.7149 (4)	0.0512 (9)	
N7	0.01317 (12)	−0.03608 (9)	0.4208 (3)	0.0398 (7)	
N8	−0.02456 (14)	−0.05705 (10)	0.3775 (4)	0.0490 (8)	
N9	−0.0244 (3)	−0.1266 (3)	0.6295 (8)	0.114 (2)	
N10	−0.1568 (2)	−0.07751 (19)	0.3548 (8)	0.0857 (18)	
C1	−0.10083 (18)	−0.03802 (13)	0.9323 (4)	0.0536 (11)	
C2	−0.1308 (2)	−0.06616 (15)	0.9360 (5)	0.0703 (15)	
H2	−0.1472	−0.0748	1.0078	0.084*	
C3	−0.13292 (19)	−0.08072 (14)	0.8077 (5)	0.0593 (12)	
C4	−0.0847 (3)	−0.01309 (18)	1.0404 (5)	0.0837 (18)	
H4A	−0.0628	0.0048	1.0073	0.126*	
H4B	−0.0695	−0.0276	1.1059	0.126*	
H4C	−0.1116	−0.0007	1.0768	0.126*	
C5	−0.1598 (2)	−0.11282 (18)	0.7556 (7)	0.0834 (18)	
H5A	−0.1533	−0.1154	0.6652	0.125*	
H5B	−0.1931	−0.1088	0.7681	0.125*	
H5C	−0.1504	−0.1349	0.8001	0.125*	
C6	−0.07031 (19)	0.06865 (11)	0.8451 (4)	0.0486 (10)	
C7	−0.11000 (19)	0.08900 (14)	0.8317 (5)	0.0606 (12)	
H7	−0.1160	0.1119	0.8692	0.073*	
C8	−0.14106 (18)	0.06886 (13)	0.7490 (5)	0.0607 (12)	
C9	−0.02660 (19)	0.07712 (13)	0.9190 (5)	0.0615 (13)	
H9A	−0.0046	0.0571	0.9103	0.092*	
H9B	−0.0344	0.0804	1.0084	0.092*	
H9C	−0.0126	0.0994	0.8862	0.092*	
C10	−0.1886 (3)	0.0808 (2)	0.7004 (8)	0.097 (2)	
H10A	−0.2016	0.0618	0.6464	0.146*	
H10B	−0.1853	0.1031	0.6515	0.146*	
H10C	−0.2094	0.0850	0.7722	0.146*	
C11	0.05422 (16)	−0.04550 (12)	0.3580 (4)	0.0465 (10)	
C12	0.0421 (2)	−0.07391 (11)	0.2775 (5)	0.0578 (12)	
H12	0.0625	−0.0868	0.2233	0.069*	
C13	−0.0061 (2)	−0.07985 (12)	0.2915 (4)	0.0569 (12)	
C14	0.10103 (19)	−0.02914 (17)	0.3787 (5)	0.0659 (14)	
H14A	0.0987	−0.0098	0.4416	0.099*	
H14B	0.1126	−0.0191	0.2989	0.099*	
H14C	0.1225	−0.0478	0.4091	0.099*	
C15	−0.0358 (3)	−0.10831 (18)	0.2264 (6)	0.092 (2)	
H15A	−0.0680	−0.1059	0.2546	0.138*	
H15B	−0.0242	−0.1325	0.2481	0.138*	
H15C	−0.0342	−0.1049	0.1347	0.138*	

### Atomic displacement parameters (Å^2^)

**Table:**

	*U*^11^	*U*^22^	*U*^33^	*U*^12^	*U*^13^	*U*^23^
Cd1	0.0606 (2)	0.05662 (19)	0.04776 (17)	−0.02047 (15)	0.00994 (15)	−0.01297 (14)
P1	0.0381 (5)	0.0323 (4)	0.0314 (4)	0.0014 (4)	0.0036 (4)	−0.0026 (3)
P2	0.0365 (6)	0.0296 (5)	0.0272 (5)	0.0004 (5)	0.000	0.000
O1	0.095 (4)	0.137 (5)	0.098 (4)	0.043 (4)	0.016 (3)	0.033 (4)
O2	0.189 (6)	0.068 (3)	0.176 (8)	−0.022 (4)	0.092 (6)	−0.016 (4)
O3	0.258 (10)	0.203 (8)	0.292 (12)	0.150 (8)	0.168 (9)	0.191 (9)
O4	0.165 (7)	0.185 (8)	0.422 (19)	−0.087 (7)	−0.175 (11)	0.126 (11)
O5	0.226 (9)	0.213 (10)	0.146 (7)	−0.137 (9)	−0.036 (7)	−0.002 (7)
O6	0.143 (6)	0.135 (5)	0.231 (9)	−0.028 (4)	−0.085 (7)	−0.035 (6)
N1	0.0366 (16)	0.0361 (15)	0.0343 (15)	−0.0021 (13)	0.0017 (12)	−0.0047 (12)
N2	0.046 (3)	0.045 (2)	0.031 (2)	0.000 (2)	0.000	0.000
N3	0.0471 (19)	0.0413 (18)	0.0403 (18)	−0.0063 (14)	0.0082 (14)	−0.0033 (14)
N4	0.0414 (18)	0.0480 (18)	0.0455 (18)	−0.0091 (16)	0.0084 (15)	−0.0042 (15)
N5	0.0419 (16)	0.0422 (16)	0.0452 (17)	0.0065 (13)	−0.0021 (16)	−0.0110 (15)
N6	0.0417 (19)	0.057 (2)	0.055 (2)	0.0120 (16)	−0.0053 (16)	−0.0146 (17)
N7	0.0434 (18)	0.0391 (17)	0.0368 (15)	0.0047 (14)	0.0064 (14)	−0.0042 (13)
N8	0.063 (2)	0.0416 (18)	0.0430 (18)	−0.0095 (17)	0.0077 (16)	−0.0141 (15)
N9	0.114 (6)	0.130 (7)	0.098 (5)	0.027 (6)	0.063 (4)	0.026 (5)
N10	0.072 (3)	0.069 (4)	0.116 (5)	−0.033 (3)	0.000 (3)	−0.008 (3)
C1	0.065 (3)	0.051 (2)	0.045 (2)	−0.009 (2)	0.017 (2)	−0.0054 (19)
C2	0.084 (4)	0.069 (3)	0.058 (3)	−0.013 (3)	0.033 (3)	0.006 (2)
C3	0.060 (3)	0.059 (3)	0.059 (3)	−0.018 (2)	0.016 (2)	0.002 (2)
C4	0.126 (5)	0.085 (4)	0.040 (3)	−0.028 (4)	0.021 (3)	−0.013 (3)
C5	0.084 (4)	0.087 (4)	0.080 (4)	−0.042 (3)	0.016 (3)	−0.003 (3)
C6	0.065 (3)	0.039 (2)	0.042 (2)	0.0061 (19)	0.002 (2)	−0.0057 (16)
C7	0.064 (3)	0.048 (3)	0.070 (3)	0.016 (2)	−0.008 (2)	−0.019 (2)
C8	0.059 (3)	0.065 (3)	0.058 (3)	0.026 (2)	0.003 (2)	−0.013 (2)
C9	0.067 (3)	0.051 (3)	0.066 (3)	−0.001 (2)	−0.004 (3)	−0.025 (2)
C10	0.078 (4)	0.099 (5)	0.114 (5)	0.039 (4)	−0.023 (4)	−0.028 (4)
C11	0.055 (3)	0.048 (2)	0.0358 (19)	0.0123 (19)	0.0070 (18)	0.0062 (17)
C12	0.079 (3)	0.053 (2)	0.042 (2)	0.013 (2)	0.012 (2)	−0.008 (2)
C13	0.086 (3)	0.044 (2)	0.040 (2)	−0.005 (2)	0.016 (2)	−0.0110 (19)
C14	0.049 (3)	0.097 (4)	0.052 (3)	0.013 (3)	0.010 (2)	−0.007 (3)
C15	0.136 (6)	0.072 (3)	0.069 (3)	−0.034 (4)	0.019 (4)	−0.036 (3)

### Geometric parameters (Å, °)

**Table:**

Cd1—N1	2.546 (3)	C1—C4	1.509 (7)
Cd1—N4	2.265 (3)	C2—C3	1.432 (8)
Cd1—N8	2.311 (4)	C2—H2	0.9300
Cd1—O1	2.509 (5)	C3—C5	1.490 (8)
Cd1—O2	2.365 (6)	C4—H4A	0.9600
Cd1—O5	2.367 (8)	C4—H4B	0.9600
Cd1—O4	2.373 (9)	C4—H4C	0.9600
P1—N2	1.576 (2)	C5—H5A	0.9600
P1—N1	1.599 (3)	C5—H5B	0.9600
P1—N5	1.650 (3)	C5—H5C	0.9600
P1—N3	1.688 (3)	C6—C7	1.349 (7)
P2—N1	1.573 (3)	C6—C9	1.485 (7)
P2—N1^i^	1.573 (3)	C7—C8	1.427 (7)
P2—N7^i^	1.693 (3)	C7—H7	0.9300
P2—N7	1.693 (3)	C8—C10	1.498 (8)
O1—N9	1.165 (11)	C9—H9A	0.9600
O2—N9	1.323 (11)	C9—H9B	0.9600
O3—N9	1.239 (10)	C9—H9C	0.9600
O4—N10	1.049 (9)	C10—H10A	0.9600
O5—N10	1.094 (12)	C10—H10B	0.9600
O6—N10	1.302 (10)	C10—H10C	0.9600
N2—P1^i^	1.576 (2)	C11—C12	1.368 (6)
N3—N4	1.372 (5)	C11—C14	1.464 (8)
N3—C1	1.376 (6)	C12—C13	1.386 (8)
N4—C3	1.328 (6)	C12—H12	0.9300
N5—N6	1.386 (5)	C13—C15	1.489 (7)
N5—C6	1.396 (5)	C14—H14A	0.9600
N6—C8	1.302 (5)	C14—H14B	0.9600
N7—C11	1.373 (5)	C14—H14C	0.9600
N7—N8	1.383 (5)	C15—H15A	0.9600
N8—C13	1.323 (6)	C15—H15B	0.9600
C1—C2	1.325 (7)	C15—H15C	0.9600
			
N4—Cd1—N8	140.76 (12)	C2—C1—N3	108.1 (4)
N4—Cd1—O2	92.8 (2)	C2—C1—C4	129.1 (5)
N8—Cd1—O2	100.51 (19)	N3—C1—C4	122.7 (4)
N4—Cd1—O5	110.4 (3)	C1—C2—C3	106.4 (4)
N8—Cd1—O5	108.2 (3)	C1—C2—H2	126.8
O2—Cd1—O5	80.4 (5)	C3—C2—H2	126.8
N4—Cd1—O4	116.7 (4)	N4—C3—C2	109.5 (4)
N8—Cd1—O4	85.8 (4)	N4—C3—C5	120.3 (5)
O2—Cd1—O4	123.8 (4)	C2—C3—C5	130.2 (5)
O5—Cd1—O4	45.7 (4)	C1—C4—H4A	109.5
N4—Cd1—O1	81.87 (16)	C1—C4—H4B	109.5
N8—Cd1—O1	77.65 (18)	H4A—C4—H4B	109.5
O2—Cd1—O1	52.5 (3)	C1—C4—H4C	109.5
O5—Cd1—O1	132.4 (4)	H4A—C4—H4C	109.5
O4—Cd1—O1	161.3 (4)	H4B—C4—H4C	109.5
N4—Cd1—N1	71.75 (11)	C3—C5—H5A	109.5
N8—Cd1—N1	72.03 (11)	C3—C5—H5B	109.5
O2—Cd1—N1	132.3 (3)	H5A—C5—H5B	109.5
O5—Cd1—N1	147.2 (4)	C3—C5—H5C	109.5
O4—Cd1—N1	103.0 (3)	H5A—C5—H5C	109.5
O1—Cd1—N1	80.3 (2)	H5B—C5—H5C	109.5
N2—P1—N1	116.61 (18)	C7—C6—N5	105.6 (4)
N2—P1—N5	108.63 (13)	C7—C6—C9	129.1 (4)
N1—P1—N5	112.25 (18)	N5—C6—C9	125.3 (4)
N2—P1—N3	110.93 (16)	C6—C7—C8	107.1 (4)
N1—P1—N3	104.34 (17)	C6—C7—H7	126.5
N5—P1—N3	103.21 (17)	C8—C7—H7	126.5
N1—P2—N1^i^	118.5 (2)	N6—C8—C7	111.0 (4)
N1—P2—N7^i^	109.24 (16)	N6—C8—C10	121.7 (5)
N1^i^—P2—N7^i^	106.30 (16)	C7—C8—C10	127.3 (4)
N1—P2—N7	106.30 (16)	C6—C9—H9A	109.5
N1^i^—P2—N7	109.24 (16)	C6—C9—H9B	109.5
N7^i^—P2—N7	106.7 (2)	H9A—C9—H9B	109.5
N9—O1—Cd1	91.9 (6)	C6—C9—H9C	109.5
N9—O2—Cd1	94.6 (5)	H9A—C9—H9C	109.5
N10—O4—Cd1	98.5 (7)	H9B—C9—H9C	109.5
N10—O5—Cd1	97.3 (6)	C8—C10—H10A	109.5
P2—N1—P1	118.8 (2)	C8—C10—H10B	109.5
P2—N1—Cd1	114.81 (15)	H10A—C10—H10B	109.5
P1—N1—Cd1	116.75 (16)	C8—C10—H10C	109.5
P1—N2—P1^i^	120.7 (3)	H10A—C10—H10C	109.5
N4—N3—C1	109.8 (3)	H10B—C10—H10C	109.5
N4—N3—P1	121.5 (3)	C12—C11—N7	105.4 (4)
C1—N3—P1	128.3 (3)	C12—C11—C14	128.2 (4)
C3—N4—N3	106.2 (4)	N7—C11—C14	126.3 (4)
C3—N4—Cd1	129.4 (3)	C11—C12—C13	107.3 (4)
N3—N4—Cd1	123.9 (2)	C11—C12—H12	126.3
N6—N5—C6	110.8 (3)	C13—C12—H12	126.3
N6—N5—P1	113.7 (3)	N8—C13—C12	111.2 (4)
C6—N5—P1	135.5 (3)	N8—C13—C15	121.0 (5)
C8—N6—N5	105.6 (4)	C12—C13—C15	127.8 (5)
C11—N7—N8	111.1 (3)	C11—C14—H14A	109.5
C11—N7—P2	131.3 (3)	C11—C14—H14B	109.5
N8—N7—P2	116.6 (2)	H14A—C14—H14B	109.5
C13—N8—N7	104.9 (4)	C11—C14—H14C	109.5
C13—N8—Cd1	125.4 (3)	H14A—C14—H14C	109.5
N7—N8—Cd1	117.8 (2)	H14B—C14—H14C	109.5
O1—N9—O3	129.7 (12)	C13—C15—H15A	109.5
O1—N9—O2	120.5 (9)	C13—C15—H15B	109.5
O3—N9—O2	109.7 (12)	H15A—C15—H15B	109.5
O4—N10—O5	118.3 (10)	C13—C15—H15C	109.5
O4—N10—O6	123.1 (10)	H15A—C15—H15C	109.5
O5—N10—O6	117.9 (8)	H15B—C15—H15C	109.5

Symmetry codes: (i) −*x*, −*y*, *z*.
